# Exploring the Efficacy and Safety of Topical Phenytoin in Androgenetic Alopecia: A Clinical Investigation

**DOI:** 10.1111/jocd.71016

**Published:** 2026-06-28

**Authors:** Sina Maskoukian, Atefeh Naeimifar, Aniseh Samadi, Hamid Akbari Javar, Afsaneh Sadeghzadeh Bazargan, Saman Ahmad Nasrollahi, Alireza Firooz

**Affiliations:** ^1^ Department of Pharmaceutical Biotechnology, School of Pharmacy Hamadan University of Medical Sciences Hamadan Iran; ^2^ Department of Pharmaceutics, Faculty of Pharmacy Tehran University of Medical Sciences Tehran Iran; ^3^ Center for Research & Training in Skin Diseases & Leprosy Tehran University of Medical Sciences (TUMS) Tehran Iran; ^4^ Department of Dermatology, Rasoul Akram Hospital Iran University of Medical Sciences (IUMS) Tehran Iran

**Keywords:** androgenetic alopecia, male pattern hair loss, phenytoin, topical solution

## Abstract

**Background:**

Phenytoin, a widely used antiepileptic drug, is known for its proliferative effects on fibroblasts and keratinocytes, as well as its stimulatory influence on angiogenesis. One of its notable side effects is hirsutism, which suggests a potential role in hair growth promotion.

**Aims:**

This study aimed to evaluate the safety and efficacy of a topical 5% phenytoin solution in men with androgenetic alopecia (AGA).

**Methods:**

In this 16‐week, open‐label clinical trial, adult male participants with AGA applied a 5% phenytoin solution twice daily. TrichoScan analysis and standardized global photographs were employed at baseline, week 8, and week 16 to assess changes in total hair count, hair density, anagen‐to‐telogen ratio, and follicular units. Subjective assessments of hair condition and scalp oiliness were obtained at each visit using a patient satisfaction scale. Adverse events were monitored throughout the study period.

**Results:**

Statistically significant improvements were observed in total hair count from baseline (158.07 ± 37.65) to week 16 (183.21 ± 44.63) (*p*‐value = 0.010). Similarly, hair density significantly increased from baseline (174.99 ± 41.70 number/cm^2^) to week 16 (202.39 ± 49.67 number/cm^2^) (*p*‐value = 0.011). Number of total follicular units also exhibited a significant increase from baseline (90.07 ± 16.85) to week 16 (98.07 ± 19.67) (*p*‐value = 0.013), as did follicular unit density from baseline (99.71 ± 18.66 FU/cm^2^) to week 16 (108.65 ± 20.94 FU/cm^2^) (*p*‐value = 0.012). The average hair unit and the anagen rate showed slight increases from baseline to week 16, but these changes were not statistically significant. Subjective assessments indicated improvements in scalp oiliness and hair texture. The treatment was well tolerated, with only mild and transient adverse events reported.

**Conclusion:**

Topical 5% phenytoin solution appears to be a safe and potentially effective therapeutic option for AGA.

## 1 Introduction

Human skin and hair play a very important role in a person's appearance and beauty. Hair loss causes many physical and psychological problems in human beings due to the decline of individual and social performance in them. Therefore, hair loss, its causes and effective treatments have always been considered [1].

Hair growth follows a cyclic pattern consisting of different stages: anagen (growth), catagen (transition), and telogen (resting). Each day, the scalp naturally sheds about 100 telogen hairs daily, while a roughly equal number of hair follicles enter the anagen phase. The length of hair is determined by the duration of the anagen phase, while the thickness is influenced by the size of the hair bulb. Factors, both systemic and local, can impact the size of hair follicles, leading to changes in the anagen phase's duration and the volume of the hair matrix. Importantly, androgens play a pivotal role in regulating hair growth. During puberty, heightened androgen levels result in follicle enlargement in areas like the beard, chest, and limbs, while concurrently reducing follicle size in the bitemporal region. This process contributes to the alteration of the hairline in men and many women [2].

There are various forms of hair loss (alopecia), one of which is AGA [3]. Androgenetic alopecia is a genetic condition sensitive to androgens, particularly dihydrotestosterone (DHT), that leads to follicular miniaturization and is the most common cause of hair loss in both men and women [4]. In men, this condition typically leads to hair loss starting in adolescence or the early twenties. This type of hair loss can be recognized by a receding hairline and the gradual reduction and thinning of hair in the head's central and frontal regions [3]. It is the most prevalent cause of hair loss [5].

Anti‐hair loss drugs include topical minoxidil [6], oral finasteride [7], anti‐androgen drugs [8], corticosteroids [9], etc., which are used to control hair loss. Some of these drugs have certain side effects; therefore, their consumption should be minimized as much as possible and alternative drugs with fewer side effects should be used.

Minoxidil is one of the drugs that is currently used topically to improve androgenic hair loss [10]. One of the important side effects of this drug is hypertrichosis. Hypertrichosis is a type of disorder of excessive hair growth and hair density, in which hair appears abnormally in all parts of the body. This condition may occur in any person, at any age. The cause behind body hair hypertrichosis remains unclear, and addressing it typically involves either physically removing the hair or discontinuing the medication to resolve the condition [11].

At therapeutic levels, phenytoin exerts its anticonvulsant impact by obstructing sodium channels and impeding the repetitive generation of action potentials. Additionally, phenytoin inhibits the release of serotonin and norepinephrine, influencing the levels of other neurotransmitters [12]. Beyond its anticonvulsant properties, phenytoin has been explored and applied in the treatment of conditions such as ulcers, epidermolysis bullosa, and inflammatory skin conditions in the field of dermatology [13], but the use of phenytoin is associated with specific allergic reactions and proliferative skin‐related side effects [14]. One of the side effects of phenytoin is hirsutism [15], which occurs on the body and face and usually ends within a year after stopping the treatment [16].

The oral anti‐hair loss properties of this medication have been investigated in a number of research. The effects of oral phenytoin on chemotherapy‐induced rat hair loss were studied, and the results showed that giving this medication to this group improved the growth and thickness of the hair, decreased the amount of skin lipid peroxidation, raised glutathione levels, and increased glutathione peroxidase activity. In a study by Onaolapo et al., it is indicated that oral phenytoin has the potential to enhance hair growth [12] and it seems that one of the mechanisms through which phenytoin may alleviate hirsutism is by its capability to suppress collagenase activity [17] and modulate the metabolism of connective tissue and the proliferation of cells in human skin fibroblast cultures [18].

In this investigation, we prepared phenytoin in the form of a topical solution because the oral form has been known to have numerous side effects and no topical form has been developed yet. Various biophysical techniques can be used to objectively and non‐invasively evaluate hair loss to evaluate the effectiveness of topical therapies. This study evaluates the effect of a topical phenytoin solution on androgenic hair loss. It is a phase IIa, proof‐of‐concept study, single‐group, before‐after clinical trial that lasted 16 weeks.

## 2 Materials and Methods

### 2.1 Materials

Phenytoin was sourced from Darou Pakhsh Pharmaceutical Co., Tehran, Iran. Methylparaben, propylparaben, and ultrapure ethanol were obtained from Merck, Germany. Deionized water was prepared in‐house as needed.

### 2.2 Preparation of Topical Solution

Phenytoin and propylparaben were dissolved in an equal ratio of deionized water to ultrapure ethanol and heated to 80°C. Subsequently, methylparaben was added, and the mixture was stirred continuously at 1000 rpm (IKA, Germany) for 5 min to yield the final solution. The formulation was then allowed to stabilize at room temperature to maintain chemical integrity.

The formulation was prepared as a clear hydroalcoholic solution. Phenytoin was solubilized using an optimized ethanol‐water ratio to ensure a homogenous phase without the requirement of salt formation, prioritizing topical tolerability and chemical stability.

### 2.3 Study Design, Participants, and Interventions

This research was a phase IIa, proof‐of‐concept study, non‐randomized, open‐blind, single group, before and after clinical trial to assess the effectiveness and safety of a topical 5% phenytoin solution in treating AGA.

This was performed in the Pharmaceutical, Cosmeceutical and Hygienic Evaluation Lab (DermaLab) of the Center of Research & Training in Skin Diseases & Leprosy (CRTSDL), Tehran University of Medical Sciences (TUMS) and Iran University of Medical Sciences (IUMS) from January 2023 to May 2023.

The study adhered to the Declaration of Helsinki, Good Clinical Practice (GCP) guidelines, local standards, ethical, and legal requirements.

The study received ethical approval from the relevant committee on December 5, 2022, with the acceptance code IR.IUMS.REC.1401.682. Furthermore, the research was registered in the Iranian Registry of Clinical Trials (IRCT) under the registration number IRCT20190901044666N3. All study participants were required to read and sign the informed consent form before their inclusion in the trial.

A total of 17 participants were enrolled in the study, and they were instructed to apply 1 mL of the topical 5% phenytoin solution twice daily to the balding area for 16 weeks. Participants were selected based on inclusion and exclusion criteria (as detailed in Table 1). Furthermore, participants were instructed to maintain their existing hairstyle, color, and length throughout the study. The 5% phenytoin topical solution was prepared by a pharmacist and stored in identical bottles, each labeled with a container number, drug dosage, date of manufacture, cautionary statements, method of administration, and storage conditions.

**TABLE 1:** Inclusion and exclusion criteria for recruiting the participants into the study.

Inclusion	Age: 18 and 50
	Gender: Male
	Class of AGA (Hamilton‐Norwood Stages): class II to IV
	Signed informed consent
Exclusion	A prior occurrence of an allergic response to phenytoin or any of the components present in the intervention solution
	Active chronic skin disease other than hair loss
	Scalp surgeries
	The habit of braiding hair
	Having any type of malignancy
	Isotretinoin use during the past year
	The use of 5‐alpha reductase inhibitors and isotretinoin within the last year, as well as the consumption of dietary supplements and herbal treatments aimed at promoting hair growth in the preceding 3 months
	Chemotherapy in the last year
	The scalp has been exposed to radiation within the last year.
	History of uncontrolled blood pressure and hypotension
	Taking systemic steroids for more than 14 days in the last 2 months

As this was an exploratory phase IIa, proof‐of‐concept study, a formal a priori power calculation was not performed.

### 2.4 Outcome Measures

The primary outcomes were changes in hair growth parameters and hair thickness from baseline to week 16. Safety evaluations comprised secondary outcomes.

### 2.5 Instrumental Measurements

All objective parameters were assessed at screening visits, weeks 8 and 16 following the intervention by:

1. Trichoscan assessment (FotoFinder Systems GmbH, Germany): The trichoscale application of FotoFinder is a novel software designed to document and quantify essential parameters related to hair growth and hair loss, including total hair density, vellus hair count, terminal hair count, average hair thickness, and the percentage of hair in the anagen and telogen phases.
2. Manual trichogram test: the percentage and frequency of hair in the anagen, telogen, catagen, and the rate of dystrophy were evaluated and recorded. These evaluations were conducted in three regions: the temporals and the vertex area, using the manual trichogram test method with Dino‐Lite device (Dino‐Lite AM4113ZT Digital Microscope, Taiwan).

### 2.6 Physician Assessment

The overall state of the hair was photographically recorded by the standard procedure. A dermatologist examined these photographs and assessed the extent of changes in the chosen area in comparison to the previous visit. The evaluation was conducted using the following scale: The number of hairs showed a significantly decrease (−2), slightly decrease (−1), no change (0), slightly increased (+1), or significantly increased (+2).

### 2.7 Patient Satisfaction

During each visit, patients were asked about their assessment of the condition of their hair in the chosen area. The evaluation was made using the following scale:

A: Worsening of hair loss (−2), Slight worsening of hair loss (−1), No change (0), Slight improvement of hair loss (+1), Extreme improvement of hair loss (+2).

B: Increase in hair oiliness (−2), Minor increase in hair oiliness (−1), No change (0), Minor reduction in hair oiliness (+1), Substantial reduction in hair oiliness (+2).

### 2.8 Safety

During the study, safety monitoring was a secondary objective. The safety population included all participants who received at least one treatment application.

The evaluation of safety involved tracking the frequency and severity of adverse events (AEs). For classification of AEs, the study employed the Common Terminology Criteria for Adverse Events (CTCAE), version 5.0 [19]. The terminology for these events was derived from the Medical Dictionary for Regulatory Activities (MedDRA Desktop Browser 4.0 Beta) [20], focusing on system organ class (SOC) and preferred term (PT).

Causality assessment for AEs was conducted using the World Health Organization–Uppsala Monitoring Centre (WHO–UMC) system [21]. Additionally, the seriousness of AEs was evaluated in accordance with the International Conference on Harmonisation E2B (ICH‐E2B) guidelines [22].

AEs detection involved participant interviews and observation of signs or symptoms during the 8th and 16th weeks of the trial.

### 2.9 Statistical Analyses

Statistical analysis in pre and post‐treatment data was analyzed using a paired‐sample *t*‐test in SPSS (version 20.0, Chicago, IL, USA), with a significance threshold of *p* < 0.05.

## 3 Results

**FIGURE 1:**
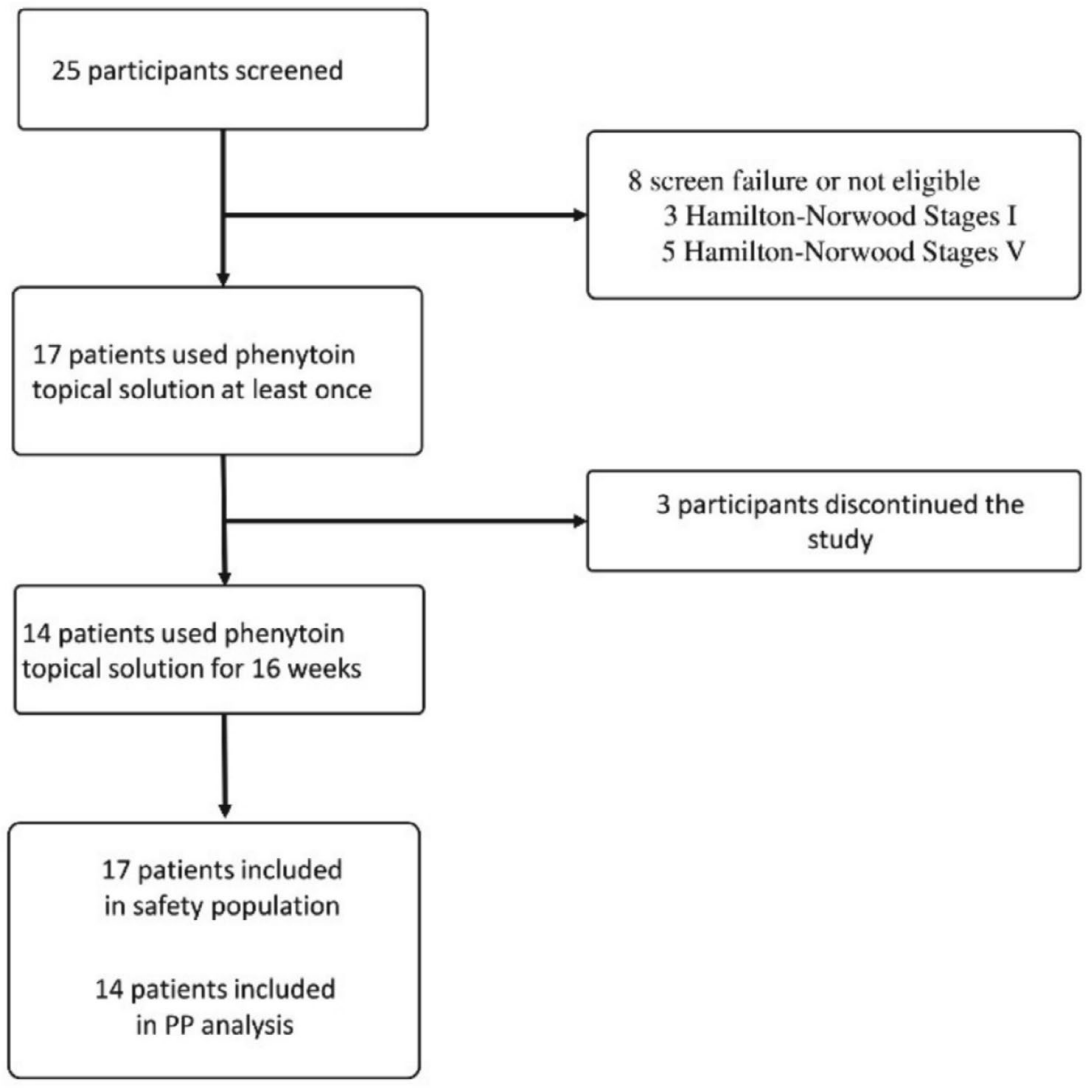
Reporting trials diagram showing the participants' flow through trial screening, taking medication, and analysis steps.

The trial was initiated on 27 January 2023. Seventeen men with androgenetic alopecia enrolled and 14 participants (82%) completed the study. All three participants withdrew from the study due to non‐compliance with the treatment regimen. Figure 1 shows the flow report of clinical trial participants. The demographic and basic characteristics of the participants are presented in Table 2.

**TABLE 2:** Participant demographics and baseline characteristics.

Characteristic	Phenytoin group (*N* = 14)
	*N* (%)
Sex (male), *n* (%)	14 (100)
Age (years)	39.92 ± 9.91
Norwood Stage II, *n* (%)	3 (21.42)
Norwood Stage III, *n* (%)	4 (28.57)
Norwood Stage IV, *n* (%)	7 (50)

### 3.1 Instrumental Measurements

#### 3.1.1 Trichoscan Assessment

**FIGURE 2:**
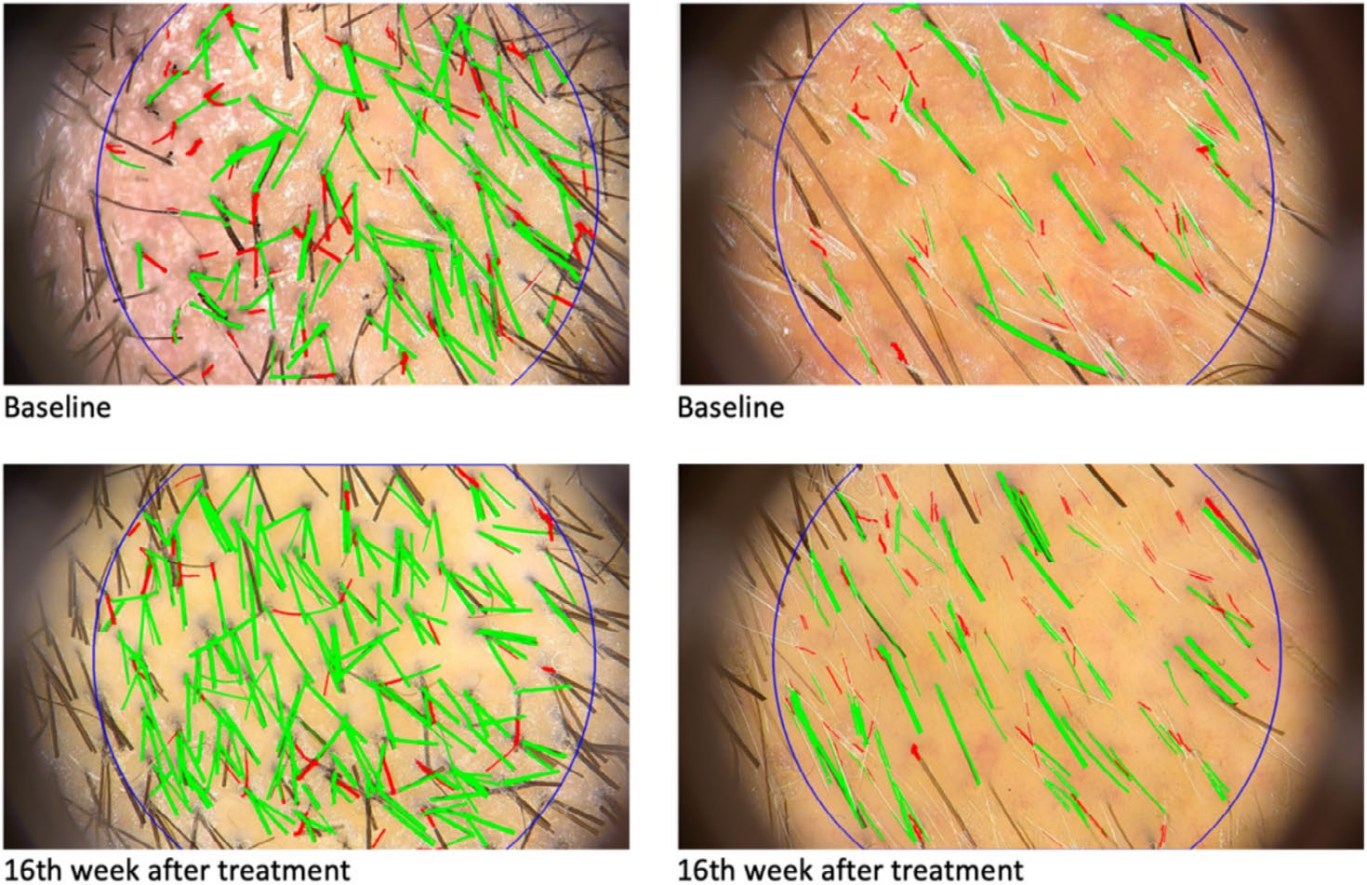
Changes in total hair count of two patients treated with topical solution of phenytoin 5%.

This method revealed that 16 weeks after therapy, phenytoin considerably enhanced the total hair count (Figure 2), hair density, total number of follicular units, and density of follicular units. Although the anagen rate and average hair unit slight increased with not statistically significant. More details of these cases are listed in Table 3.

**TABLE 3:** Trichoscale parameters at the baseline and visit 1 and 2.

Parameters	Baseline	Week 8	Week 16	*p* *	*p* **
	Mean ± SD	Mean ± SD	Mean ± SD		
*Trichoscale assessment*					
Total hair count (number)	158.07 ± 37.65	161.71 ± 36.49	183.21 ± 44.63	0.387	**0.010**
Hair density (number/cm^2^)	174.99 ± 41.70	179.03 ± 40.41	202.39 ± 49.67	0.386	**0.011**
Hair thickness (mm)	0.06 ± 0.01	0.062 ± 0.009	0.06 ± 0.01	0.571	0.929
Anagen rate (%)	68.96 ± 8.40	67.14 ± 12.51	70.55 ± 9.26	0.496	0.518
Telogen rate (%)	31.04 ± 8.41	32.85 ± 12.51	29.45 ± 9.25	0.497	0.518
Anagen/Telogen ratio	2.42 ± 0.84	2.48 ± 1.35	2.42 ± 0.84	0.849	0.121
Terminal rate (%)	79.09 ± 13.14	80.69 ± 12.83	75.98 ± 16.84	0.392	0.368
Vellus rate (%)	20.9 ± 13.14	19.3 ± 12.83	24.01 ± 16.84	0.392	0.368
Terminal/Vellus ratio	7.41 ± 10.7	6.05 ± 3.81	4.73 ± 3.16	0.632	0.355
Total Follicular units	90.07 ± 16.85	91.42 ± 14.66	98.07 ± 19.67	0.580	**0.013**
Follicular units density (FU/cm^2^)	99.71 ± 18.66	101.21 ± 16.23	108.65 ± 20.94	0.579	**0.012**
Average hair unit	1.74 ± 0.17	1.75 ± 0.13	1.85 ± 0.14	0.779	0.090

*Note:* The underlined bold values indicate statistically significant differences.

* Comparison between baseline and after 8 weeks of therapy (Wilcoxon rank test).

** Comparison between baseline and after 16 weeks of therapy (Wilcoxon rank test).

#### 3.1.2 Manual Trichogram Test

Based on the manual trichogram method, the anagen rate in week 16 increased slightly compared to the baseline, but it was not significant. The telogen rate decreased after 16 weeks, which was also not significant. More details of these cases are listed in Table 4.

**TABLE 4:** Trichogram parameters at the baseline and visit 1 and 2.

Parameters (%)	Baseline	8th week	16th week	*p* *	*p* **
	Mean ± SD	Mean ± SD	Mean ± SD		
*Manual trichogram test*					
Anagen rate	43.11 ± 10.63	44.77 ± 12.81	48.34 ± 15.21	0.450	0.057
Telogen rate	21.31 ± 5.46	24.81 ± 7.35	19.90 ± 7.37	0.069	0.390
Catagen rate	14.16 ± 6.48	12.45 ± 5.26	14.47 ± 7.08	0.424	0.905
Dystrophic rate	11 ± 9.54	9.25 ± 8.5	7.72 ± 7.96	0.253	0.057

* Comparison between baseline and after 8 weeks of therapy (Wilcoxon rank test).

** Comparison between baseline and after 16 weeks of therapy (Wilcoxon rank test).

### 3.2 Physician Assessment

**FIGURE 3:**
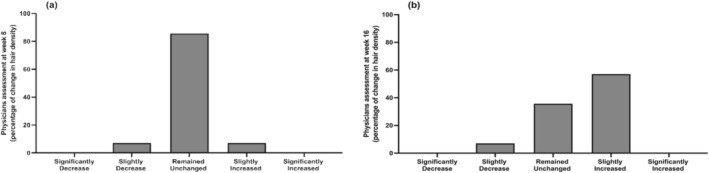
Five point rating scale used by Physicians' assessment after 8 weeks of treatment against baseline (a), Physicians' assessment after 16 weeks of treatment against baseline (b).

Hair density was assessed at 8 and 16 weeks post‐intervention based on dermatologist evaluations. After 8 weeks of treatment, 7.1% of participants experienced a slight increase in hair density, while 85.7% showed no change, and 7.1% experienced a slight decrease (Figure 3a). By week 16, hair density slightly increased in 57.1% of participants, with 35.7% showing no change and 7.1% experiencing a slight decrease (Figure 3b).

### 3.3 Patient Satisfaction

**FIGURE 4:**
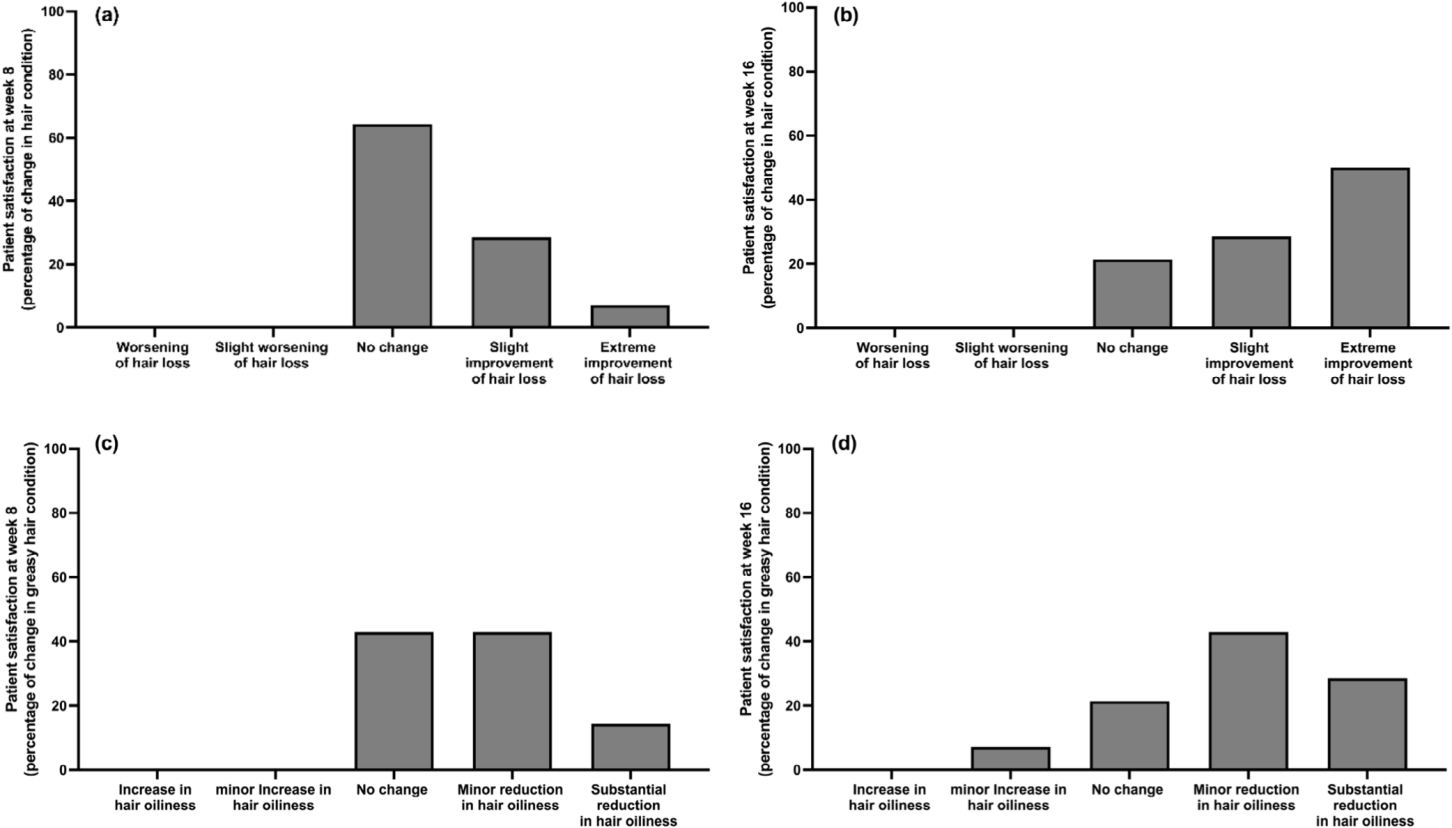
Five point rating scale used by patients' satisfaction for change in hair condition after 8 weeks of treatment against baseline (a), Patients' satisfaction for change in hair condition after 16 weeks of treatment against baseline (b), Patients' satisfaction for grassy hair condition after 8 weeks of treatment against baseline (c), Patients' satisfaction for grassy hair condition after 16 weeks of treatment against baseline (d).

Sensory evaluation of hair loss was conducted at the 8th and 16th weeks. After 8 weeks, 64.3% participants reported no change, 28.6% noted slight improvement of hair loss, and 7.1% reported extreme improvement of hair loss (Figure 4a). By week 16, 21.4% reported no change, 28.6% noted slight improvement of hair loss, and 50% reported extreme improvement of hair loss compared to baseline (Figure 4b).

Similarly, sensory evaluation of hair oiliness was assessed. After 8 weeks, 42.9% reported no change, 42.9% reported a minor reduction in hair oiliness, and 14.3% reported a substantial reduction in hair oiliness (Figure 4c). By week 16, 7.1% reported a minor increase in hair oiliness, 21.4% reported no change, and 71.5% reported a minor or substantial reduction in hair oiliness (Figure 4d).

### 3.4 Safety

In this study, safety was examined in 17 patients, and 9 AEs were reported. The frequency of all AEs was reported based on the PT and SOC. Besides, AEs with grade 3 or higher and serious AEs were not observed in this study. The frequency of AEs according to SOC and PT is shown in Table 5.

**TABLE 5:** Frequency of AEs classified by SOC and PT.

System organ class	Preferred term name	Phenytoin solution
		*N* = 17
Number of AEs		9 (100)
Skin and subcutaneous tissue disorders	At least one event	7 (77.78)
	Rash	4 (44.45)
	Pruritus	2 (22.22)
	Skin inflammation	1 (11.11)
Nervous system disorders	At least one event	2 (22.22)
	Headache	2 (22.22)

*Note:* Data in this table is presented as frequency (%).

Details of causality assessments for AEs are provided in Table 6. The causality of six (66.67%) AEs was assessed as probable.

**TABLE 6:** Frequency of AEs classified by causality assessment results.

Causality	Phenytoin solution
	Number of AEs = 9
Probable/Likely	6 (66.67)
Possible	1 (11.11)
Unlikely	2 (22.22)

## 4 Discussion

This phase IIa clinical trial demonstrated that topical application of a 5% phenytoin solution over a 16‐week period led to significant improvements in objective hair growth parameters, including total hair count, hair density, and follicular unit density in men with AGA. These findings, supported by favorable subjective satisfaction scores and minimal adverse events, suggest that phenytoin may serve as a promising alternative or adjunctive treatment for AGA, particularly in patients who are unresponsive or intolerant to conventional therapies like minoxidil and finasteride.

The observed clinical improvements can be mechanistically justified through several well‐established biological pathways. Hirsutism refers to the development of terminal, coarse, and pigmented hair in regions associated with sexual hair growth [23].

One of the side effects of phenytoin drug is hirsutism [15], which has been identified in various studies on the anti‐hair loss effects of this drug taken orally [12]. Elevated hair growth has been documented in approximately 8%–12% of individuals undergoing phenytoin treatment, typically manifesting within the initial 2 to 3 months of therapy [24]. Phenytoin has various effects on the levels of magnesium, zinc, and copper present in hair [18] and in another study by Onaolapo et al. at 2010, it was observed that phenytoin can be effective in increasing capillary phase and blood flow [25]. These properties make phenytoin a good candidate for preparing a topical dosage form.

There were only a few early studies that evaluated the effect of oral phenytoin in AGA, so this article investigated whether the results of previous studies could be confirmed in clinical conditions in vivo and with topical formulations.

In a study conducted by Onaolapo et al. [12], they explored the impact of oral phenytoin on hair growth in rats undergoing cyclophosphamide (CYP) treatment, with the objective of assessing its potential to counteract chemotherapy‐induced hair loss. Phenytoin was administered in conjunction with CYP. The results ultimately demonstrated that the concurrent use of phenytoin was linked to improved hair growth, increased thickness of hair shafts, reduced skin lipid peroxidation, elevated levels of glutathione, and increased glutathione peroxidase activities. This research concluded that oral phenytoin has the ability to mitigate hair loss resulting from CYP treatment in rats [12].

In a case report, it is reported that a 9‐year‐old boy complained of hair growth all over his body during 1 year, suffered from generalized epilepsy and received treatment for it, which included a combination of antiepileptic medications, including a 50 mg dose of phenytoin taken three times daily from the time of diagnosis. He exhibited hair growth throughout his entire body, with the most noticeable areas being his back, buttocks, arms, and thighs. Finally, it was found that the above cases refer to hypertrichosis caused by phenytoin, and after stopping this drug, the symptoms of hypertrichosis almost disappeared after 1 month [26].

Recent mechanistic insights suggest that phenytoin may exert hair growth‐promoting effects through multiple biological pathways. Firstly, it activates the gp130‐JAK‐STAT3 axis, enhancing the expression of vascular endothelial growth factor (VEGF) and transforming growth factor beta‐1 (TGF‐β1), which are key regulators of angiogenesis and dermal remodeling [27]. Secondly, phenytoin increases the levels of epidermal growth factor (EGF) and VEGF, promoting keratinocyte proliferation and extracellular matrix regeneration, both essential for follicular health [28]. Thirdly, it inhibits collagenase activity, thereby preserving the structural integrity of the perifollicular matrix, which supports hair follicle anchorage [17]. Fourthly, phenytoin exhibits potent antioxidant effects by upregulating superoxide dismutase (SOD) and glutathione peroxidase (GSH‐Px). This upregulation mitigates oxidative stress—a major contributor to hair follicle miniaturization and cycling disruption, which is followed by phenytoin may reduce apoptosis and promote anagen entry, as evidenced by increased follicular density and synchronized growth patterns in animal models [12]. Lastly, administration of phenytoin has been shown to have a stimulatory effect on connective tissue remodeling and angiogenesis, which may provide trophic support for damaged hair follicles. These multifaceted actions collectively position phenytoin as a biologically plausible agent for treating androgenetic alopecia.

The current study involved a before‐after assessment of the impact of a 5% phenytoin solution on various parameters, including total hair count, hair density, total follicular units, and follicular units density, and the percentage of hair in the anagen and telogen phases.

The comparison between the baseline level and the values 16 weeks after phenytoin consumption was considered as the efficacy and safety outcomes of the study.

Following 16 weeks of treatment, there was a notable statistically difference in total hair count, Hair density, total Follicular units, and Follicular units density before and after treatment with phenytoin.

In this study, phenytoin increased the vellus hair count. Increase in total hair density subsequently depends on the increase in vellus hair count. This finding lends credence to the notion that, initially, phenytoin stimulates the production of vellus hairs, which subsequently transition into terminal hairs with ongoing treatment, and it may be indicated that in the early stages the augmentation in total hair density may be attributed more to the development of vellus hair rather than terminal hair.

Safety and tolerability are key concerns in topical therapies. In our study, the adverse events were mild and transient, with adverse reactions, such as redness and itching, observed in our study, alongside no severe adverse events, which underscores the topical formulation's safety profile. Additionally, the reported improvement in scalp oiliness by 71.5% of participants at week 16 signifies a potential benefit for long‐term use.

While our study presents promising results, it is limited by its small sample size and lack of a placebo‐controlled design. As a Phase IIa exploratory research, this study used a pilot sample size (*n* = 14) without an a priori power calculation. While the small cohort may limit generalizability, statistically significant improvements were observed in key parameters, demonstrating a statistically significant preliminary clinical signal. These findings provide the essential effect size data required to power future randomized controlled trials (RCTs) and should be interpreted as proof of concept. Clinically, our study suggests topical phenytoin as a potential treatment option for androgenetic alopecia, especially for patients seeking alternative treatments. However, future research should focus on larger, randomized controlled trials to validate the efficacy and safety of topical phenytoin in treating androgenetic alopecia and pharmacokinetic assessments. Investigating the molecular mechanisms underlying phenytoin's effect on hair growth and exploring its combination with other treatments could offer new insights. Studies examining long‐term use and comparing phenytoin directly with established treatments like minoxidil and finasteride would further clarify its position in hair loss management strategies.

## 5 Conclusion

This study demonstrated that topical application of a 5% phenytoin solution significantly improved multiple hair growth parameters—including total hair count, hair density, and follicular unit density in men with AGA over a 16‐week period. The formulation was well tolerated, with no severe adverse events reported, and most participants reported subjective improvements in both hair condition and scalp oiliness. Initial findings from a before‐and‐after evaluation showed promising results for the use of topical phenytoin in treating androgenic hair loss. However, further validation through larger, randomized, placebo‐controlled trials is essential to confirm its efficacy and long‐term safety, and to determine its place within the current treatment paradigm.

## Author Contributions

Afsaneh Sadeghzadeh Bazargan conceived the study idea, provided clinical supervision, and evaluated patients in collaboration with Alireza Firooz. The study was designed by Afsaneh Sadeghzadeh Bazargan, Saman Ahmad Nasrollahi, Alireza Firooz, and Hamid Akbari Javar. Data collection was performed by Sina Maskoukian, Aniseh Samadi, and Atefeh Naeimifar. Sina Maskoukian and Atefeh Naeimifar were responsible for the preparation of the drug formulation, under the scientific supervision of Hamid Akbari Javar and Saman Ahmad Nasrollahi, who also oversaw stability testing. Clinical trial execution was carried out by Sina Maskoukian and Aniseh Samadi. The first draft of the manuscript was prepared by Sina Maskoukian, and the final version was reviewed and edited by Afsaneh Sadeghzadeh Bazargan, Saman Ahmad Nasrollahi, and Alireza Firooz. All authors contributed to the interpretation of the findings, reviewed the manuscript critically for important intellectual content, and approved the final version for submission.

## Ethics Statement

The study adhered to the Good Clinical Practice (GCP) guidelines and the Declaration of Helsinki in conducting the research. The Iran University of Medical Sciences' Research Ethics Committee granted approval for the study, with the reference number IR.IUMS.REC.1401.682. The trial was registered in the Iranian Registry of Clinical Trial (IRCT) under the code IRCT20190901044666N3, with the registration date being 17/12/2022.

## Consent

Prior to commencing the trial, every participant provided their signature on a written informed consent document.

## Conflicts of Interest

The authors declare no conflicts of interest.

## Data Availability

The data that support the findings of this study are available from the corresponding author upon reasonable request.
